# Impact of coronavirus disease 2019 (COVID-19) pandemic on patients with migraine: a web-based survey study

**DOI:** 10.1186/s10194-020-01183-6

**Published:** 2020-09-24

**Authors:** Jasem Y. Al-Hashel, Ismail Ibrahim Ismail

**Affiliations:** 1 Department of Neurology, Ibn Sina Hospital, Sabah Medical Region, Kuwait; 2 Department of Medicine, Health Sciences Centre, Kuwait University, Jabriya, Kuwait

**Keywords:** Coronavirus, COVID-19, Pandemic, Migraine, Psychosocial, Treatment

## Abstract

**Background:**

Since the declaration COVID-19 as a pandemic, healthcare systems around the world have faced a huge challenge in managing patients with chronic diseases. Patients with migraine were specifically vulnerable to inadequate medical care. We aimed to investigate the “real-world” impact of COVID-19 pandemic on migraine patients, and to identify risk factors for poor outcome.

**Methods:**

We administered an online, self-reported survey that included demographic, migraine-related, COVID-19-specific and overall psychosocial variables between July 15 and July 30, 2020. We recruited a sample of patients with migraine from headache clinic registry and via social media to complete an anonymous survey. Outcomes included demographic variables, change in migraine frequency and severity during the lockdown period, communication with treating physician, compliance to migraine treatment, difficulty in getting medications, medication overuse, symptoms of anxiety and/or depression, sleep and eating habits disturbance, screen time exposure, work during pandemic, use of traditional medicine, effect of Botox injection cancellation, and overall worries and concerns during pandemic.

**Results:**

A total of 1018 patients completed the survey. Of the respondents, 859 (84.3%) were females; 733 (71.9%) were aged 20 to 40 years, 630 (61.8%) were married, and 466 (45.7%) reported working during the pandemic.

In comparison to pre-pandemic period, 607 respondents (59.6%) reported increase in migraine frequency, 163 (16%) reported decrease in frequency, and 105 (10.3%) transformed to chronic migraine. Severity was reported to increase by 653 (64.1%) respondents. The majority of respondents; 626 (61.5%) did not communicate with their neurologists, 477 (46.9%) reported compliance to treatment, and 597 (58.7%) reported overuse of analgesics. Botox injections cancellation had a negative impact on 150 respondents (66.1%) from those receiving it. Forty-one respondents (4%) were infected with COVID-19; 26 (63.4%) reported worsening of their headaches amid infection period. Sleep disturbance was reported by 794 (78.1%) of respondents, and 809 (79.5%) reported having symptoms of anxiety and/or depression.

**Conclusions and relevance:**

COVID-19 pandemic had an overall negative impact on patients with migraine. Several risk factors for poor outcome were identified. Long-term strategies should be validated and implemented to deliver quality care for patients with migraine, with emphasis on psychosocial well-being.

## Background

Severe acute respiratory syndrome coronavirus 2 (SARS-CoV-2) emerged in Wuhan, China in December 2019, and coronavirus disease 2019 (COVID-19) was declared a pandemic in March 11, 2020 [1]. Since then, health care systems around the world have been overwhelmed with this unprecedented public health crisis. Neurologists are facing a huge challenge in providing quality care to their patients with chronic diseases, in particular those with migraine, while working to minimize the spread of COVID-19 outbreak [2].

Migraine is considered one of the most disabling chronic neurological diseases, and patients with migraine are particularly vulnerable to drastic negative impacts of the pandemic. From heightened levels of psychosocial stress, social isolation, disruption of sleep and dietary habits, to several COVID-19-specific concerns, patients with migraine can suffer from losing their earlier therapeutic response, especially with cancellation of face-to-face visits and procedural treatments [3]. Headache units all over the world have tried to adapt to the situation by implementing new approaches such as phone calls or Internet applications. Unfortunately, not all countries were capable of delivering the same quality care, and in many cases, this has led to deterioration in patient management [4].

Migraine is highly prevalent in Kuwait. In a recent population-based study [5] of 15,523 subjects, episodic and chronic migraine had a prevalence of 28.5%, compared to around 10% worldwide prevalence [6, 7].

The “real-world” impact of COVID-19 pandemic on patients with migraine has not been studied, to the best of our knowledge. In this cross-sectional study, we aim to investigate this impact on migraine frequency and severity, medical care, doctor-patient communication, in addition to overall psychosocial health and specific COVID-19 concerns in Kuwait.

## Methods

### Participants

This cross-sectional, internet-based study, recruited patients with migraine between July 15 and July 30, 2020, using an online free, open access “Google™ Forms” survey.

The study was approved by the Institutional Review Board Committee of Ministry of health of the state of Kuwait.

The survey investigated demographic variables, frequency and severity of migraine attacks, patient’s ability to get proper medical care, symptoms of depression, anxiety, insomnia, perceived stress, analgesics overuse, the use of other traditional/alternative remedies, and patient’s COVID-19-specific concerns during the pandemic.

Participants were recruited from 2 sources; (1) patients with migraine who are registered in headache outpatient clinic at Ibn Sina Hospital, the largest neurology tertiary center in Kuwait, and (2) through sharing the survey via two medical social media accounts in Kuwait, with participants who have received a medical diagnosis of migraine prior to the pandemic only included.

The survey continued until there was no more responses for 1 day. The survey was closed at 23:59, July 30, 2020.

### Survey

The survey contained twenty-five self-administered questions, comparing different variables in the 3 months period prior to, and during the social lockdown. The lockdown in Kuwait started at midnight of March 11, 2020. The questionnaire covered the following items:

(1) Socio-demographic variables (age, gender, educational level, marital status, occupation, working during the pandemic). (2) Migraine related variables (onset, medical/ procedural treatments, emergency department visits). (3) Changes in headache frequency during COVID-19 pandemic (number of days before and during lockdown). (4) Changes in headache severity during COVID-19 pandemic (slight-moderate-significant). (5) Medications related variables (availability of the drugs, compliance to treatment, medications overuse, OnabotulinumtoxinA (BOTOX®-Allergan) injection, use of alternative/traditional medicine). (6) Associated sleep and dietary disturbances. (7) Associated symptoms for depression and/or anxiety. (8) COVID-19-related variables (infection with the virus, specific worries and concerns during the pandemic, screen time exposure, and exercise during the pandemic).

In this survey, the main outcome variable was change in migraine frequency and severity during the pandemic, in comparison to the 3 months pre-pandemic period. We aim to explore risk factors associated with worsening of headache frequency and severity.

The survey link was created and sent through WhatsApp application to patients with migraine registered in headache clinic. It was also posted on two medical social media accounts in Kuwait (including Instagram and Facebook). It was available in two languages (Arabic -English). No personally identifiable information was collected. Answering the survey was considered an implied consent to participate in the study, as approved by Institutional Review Board Committee, based on the fact that completing the survey was voluntary, and all answers were confidential.

### Statistical analysis

Data analysis was performed from August 1, 2020, to August 5, 2020. Data were analyzed using SPSS statistical software version 25.0 (Armonk, NY: IBM Corp). The Kolmogorov- Smirnov test was used to verify the normality of distribution of variables; Comparisons between groups for categorical variables were assessed using Chi-square test (Fisher or Monte Carlo). Mann-Whitney test was used to compare between two groups for not normally distributed quantitative variables.

Univariate logistic regression was used to determine the association between the different studied variables and change in headache frequency and severity. All of the variables that were statistically significant were entered into multivariate logistic regression model to explore the factors that are independently associated with migraine worsening. The multivariate model contained variables that were associated with *p* < 0.1 in univariate analysis. Significance of the obtained results was set at *p* < 0.05 level.

## Results

### Demographic data

A total of 1018 patients completed the survey; 432 (42.4%) from the headache outpatient registry and 586 (57.6%) through the social media responses. The response rate could not be calculated in this study.

Of the respondents, 859 (84.3%) were females; 733 (71.9%) were aged 20 to 40 years, 236 (23.2%) were aged 40 to 60 years, 630 (61.8%) were married, 527 (51.7%) had a full-time job, 456 (44.8%) had high school level education, and mean disease duration was 9.8 ± 8.3 years. During the pandemic, 465 (45.7%) reported working, either from home, or as essential workers. Baseline characteristics of participants are listed in Table 1.

**Table 1:** Distribution of survey respondents according to different variables (*n* = 1018)

	No. (%)
**Age (years)**	
< 20	38(3.7%)
20–40	733(72%)
40–60	235(23.1%)
> 60	12 (1.2%)
**Gender**	
Female	858 (84.3%)
Male	160 (15.7%)
**Occupation**	
Full-time job	526 (51.7%)
I don’t work	208 (20.4%)
Student	116 (11.4%)
Part-time job	107 (10.5%)
Retired	61 (6%)
**Marital status**	
Married	629 (61.8%)
Single	324 (31.8%)
Divorced	55 (5.4%)
Widow	4 (0.4%)
Other	6 (0.6%)
**Education**	
High school	456 (44.8%)
Primary school or less	282 (27.7%)
University	280 (27.5%)
**Disease onset (years)**	
Mean ± SD.	9.8 ± 8.3
Median (Min. – Max.)	7 (1–46)
**Increase in Migraine severity**	653 (64.1%)
**Increase in Migraine frequency**	607 (59.6%)
**Degree of severity increase**	
Did not increase	365 (35.9%)
Moderate increase	357 (35.1%)
Significant increase	231 (22.7%)
Slight increase	65 (6.4%)
**Migraine days (before pandemic)**	
Mean ± SD.	5.7 ± 5.5
Median (Min. – Max.)	4 (0–30)
**Migraine days (during pandemic)**	
Mean ± SD.	8 ± 7.1
Median (Min. – Max.)	5 (0–30)
**Decrease in migraine days**	163 (16%)
**Medication overuse**	597 (58.7%)
**Compliance to migraine treatment**	477 (46.9%)
**Difficulty in getting medications**	256 (25.1%)
**Emergency department visit**	228 (22.4%)
**Chronification of migraine during pandemic**	105 (10.3%)
**Communication with treating physician**	
Did not contact for the duration of the pandemic	626 (61.5%)
Visiting the doctor / hospital	136 (13.4%)
Telephone	100 (9.8%)
Social media	98 (9.6%)
Other	38(3.7%)
Telemedicine clinics	20 (2%)
**Worries and concerns during pandemic**	
Infection with COVID-19	571 (56.1%)
Increase migraine severity and/or frequency	445 (43.7%)
Unavailability of medications	174 (17.1%)
Social, financial and family problems	175 (17.1%)
Losing their jobs	50 (4.9%)
Other concerns	136 (13.4%)
**Sleep disturbance**	794 (78.1%)
**Increase screen time exposure**	905 (88.9%)
**Symptoms of anxiety and/or depression**	809 (79.5%)
**Eating habits disturbance**	593 (58.3%)
**Working during pandemic**	465 (45.7%)
**Use of alternative/ traditional medicine**	406 (39.9%)
**Lack of regular exercise**	812 (79.7%)
**Negative effect of Botox injection cancellation**	
No	77 (33.9%)
Yes	150 (66.1%)
**COVID-19 infection**	41 (4%)
**Headache worsening during COVID-19 infection**	
No	15 (36.6%)
Yes	26 (63.4%)

### Migraine-related data

In comparison to pre-pandemic period, 607 respondents (59.6%) reported increase in migraine frequency, with mean numbers of attacks before and during the pandemic are (5.7 ± 5.5) and (8 ± 7.1) days per month, respectively. Of them, 105 respondents (10.3%) reported having 15 or more days of headache per month for more than 3 months during the pandemic, fulfilling the diagnostic criteria of chronic migraine. Another 163 (16%) reported decrease in headache frequency.

Migraine severity was reported to increase by 653 respondents (64.1%) with 65 (6.4%) describing this increase as slight, 357 (35.1%) as moderate, and 231(22.7%) as significant. Among them, 229 (22.5%) reported having very severe attacks necessitating emergency department (ED) visits. The majority of respondents; 626 (61.5%) did not communicate with their neurologists during the lockdown period. Of those who communicated; 136 patients (13.4%) reported visiting the hospital, 100 patients (9.8%) used telephone, 98 patients (9.6%) used social media, 20 patients (2%) used telemedicine, and 38 patients (3.7%) used other methods.

As regard to medication-related questions; 256 respondents (25.1%) reported having difficulty in getting their medications, 477 (46.9%) reported compliance to treatment, 597 (58.7%) reported overuse of analgesics, and 406 (39.9%) reported using traditional/ alternative regimens during the pandemic.

Of those receiving Botox injections, 150 (66.1%) reported negative impact of procedure cancellation. Forty-one respondents (4%) were infected with COVID-19. Of them; 26 (63.4%) reported worsening of their headaches amid infection period.

### Psychosocial data

Sleep disturbance was reported by 794 (78.1%) of respondents, disturbance of eating habits by 593 (58.3%), and 809 (79.5%) reported having symptoms of anxiety and/or depression.

The majority of respondents reported increased screen time exposure; 905 (88.9%), and lack of regular exercise; 812 (79.7%).

The main worries and concerns of the respondents were; infection with COVID-19 in 571(56.1%), increase migraine severity and/or frequency in 445 (43.7%), social, financial and family problems in 175 (17.1%), unavailability of medications in 174 (17.1%), losing their job in 50 (4.9%), and other concerns in 136 (13.4%).

### Correlations

There was a statistically significant correlation between increase migraine frequency and female gender (*p* = 0.004), shorter disease duration (mean duration: 8.4 ± 8.7 years vs 10.3 ± 7.4, *p* = 0.031), difficulty in getting medications (179 patients (29.5%) vs 77 patients (18.7%), *p* = < 0.001), non-compliance to migraine treatment (330 patients (54.4%) vs 147 patients (35.8%), *p* = < 0.001), medication overuse (497 patients (81.9%) vs 100 patients (24.3%), *p* = < 0.001), lack of communication with treating physician (425 patients (70.0%) vs 201 patients (48.9%), *p* = < 0.001), associated sleep disturbance (535 patients (88.1%) vs 259 patients (63%) vs, *p* = < 0.001), eating habits disturbance (418 patients (68.9%) vs 175 patients (57.4%), *p* = < 0.001), symptoms of anxiety and/or depression (523 patients (86.2%) vs 285 patients (69.3%), *p* = < 0.001), working during pandemic (295 patients (48.5%) vs 170 patients (41.3%), *p* = 0.042), use of alternative/ traditional medicine (270 patients (44.5%) vs 136 patients (33.1%), *p* = < 0.001), and lack of regular exercise (544 patients (66.9%) vs 268 patients (33.1%), *p* = < 0.001). Furthermore, migraine severity showed a statistically significant correlation with the same variables. However, there was no statistically significant correlation between worsening of migraine frequency and severity with age, occupation, education, marital status, and screen time exposure. Detailed findings are summarized in Table 2.

**Table 2:** Relation between increase in migraine frequency and increase in migraine severity with different variables (n = 1018)

Variables	Total No.	Increase in migraine days (***n*** = 607)	Test of Sig.	***p***-value	Increase in migraine severity (***n*** = 653)	Test of Sig.	***p***-value
**Age (years)**							
< 20	**38**	22 (3.6%)	χ^2^ = 0.528	0.913	29 (4.5%)	χ^2^ = 3.192	0.363
20–40	**733**	438 (72.2%)			469 (72.2%)		
40–60	**235**	141 (23.2%)			145 (22.3%)		
> 60	**12**	6 (1%)			7 (1.1%)		
**Gender**							
Male	**858**	79 (13%)	χ^2^ = 8.288^*^	0.004^*^	82 (12.6%)	χ^2^ = 13.059^*^	< 0.001^*^
Female	**160**	528 (87%)			568 (87.4%)		
**Occupation**							
Not working	**208**	112 (18.5%)	χ^2^ = 8.702	0.069	124 (19.1%)	χ^2^ = 3.971	0.563
Student	**116**	60 (9.9%)			73 (11.2%)		
Part time job	**107**	67 (11%)			69 (10.6%)		
Full time job	**526**	331 (54.5%)			347 (53.4%)		
Retired	**61**	37 (6.1%)			37 (5.7%)		
**Marital status**							
Single	**324**	185 (30.5%)	χ^2^ = 6.315	0.163	204 (31.4%)	χ^2^ = 2.057	0.748
Married	**629**	386 (63.6%)			403 (62.0%)		
Widow	**4**	4 (0.7%)			4 (0.6%)		
Divorced	**55**	30 (4.9%)			35 (5.4%)		
Other	**6**	2 (0.3%)			4 (0.6%)		
**Education**							
High school	**456**	215 (47.1%)	χ^2^ = 5.413	0.179	248 (54.3%)	χ^2^ = 5.688	0.099
Primary school or less	**282**	143 (50.7%)			136 (48.2%)		
University	**280**	161 (57.4%)			144 (51.4%)		
**Disease onset**							
Mean ± SD.		8.4 ± 8.7	U = 114,845.0^*^	0.031^*^	8.5 ± 8.2	U = 107,303.50^*^	0.006^*^
Median (Min. – Max.)		6 (1–46)			6 (1–46)		
**Difficulty in getting medications**	**256**	179 (29.5%)	χ^2^ = 15.058^*^	< 0.001^*^	190 (29.2%)	χ^2^ = 15.928^*^	< 0.001^*^
**Compliance to migraine treatment**							
No	**541**	277 (45.6%)	χ^2^ = 34.045^*^	< 0.001^*^	305 (46.9%)	χ^2^ = 27.940^*^	< 0.001^*^
Yes	**477**	330 (54.4%)			345 (53.1%)		
**Medication overuse**	**597**	497 (81.9%)	χ^2^ = 334.634^*^	< 0.001^*^	504 (77.5%)	χ^2^ = 264.669^*^	< 0.001^*^
**Communication with treating physician**							
Did not contact the doctor for the duration of the pandemic	**626**	425 (70.0%)	χ^2^ = 66.015	< 0.001^*^	471 (72.1%)	χ^2^ = 110.693	< 0.001^*^
Contacted treating physician	**392**	182 (30.0%)			182 (27.9%)		
**Sleep disturbance**	**794**	535 (88.1%)	χ^2^ = 90.115^*^	< 0.001^*^	562 (86.5%)	χ^2^ = 75.083^*^	< 0.001^*^
**Eating habits disturbance**	**593**	418 (68.9%)	χ^2^ = 69.618^*^	< 0.001^*^	443 (68.2%)	χ^2^ = 72.501^*^	< 0.001^*^
**Symptoms of anxiety and/or depression**	**809**	523 (86.2%)	χ^2^ = 42.337^*^	< 0.001^*^	561 (86.3%)	χ^2^ = 52.838^*^	< 0.001^*^
**Increase screen time exposure**	**905**	545 (89.8%)	χ^2^ = 1.196	0.274	584 (89.8%)	χ^2^ = 1.632	0.201
**Working during pandemic**	**465**	295 (48.5%)	χ^2^ = 3.522^*^	0.042^*^	312 (48.0%)	χ^2^ = 3.908^*^	0.039^*^
**Use of alternative/ traditional medicine**	**406**	270 (44.5%)	χ^2^ = 13.263^*^	< 0.001^*^	283 (43.5%)	χ^2^ = 10.026^*^	0.002^*^
**Lack of regular exercise**	**812**	544 (66.9%)	χ^2^ = 12.263^*^	< 0.001^*^	498 (61.3%)	χ^2^=10.405^*^	< 0.001^*^

χ^2^: Chi square test U: Mann Whitney test

p: *p* value for association between different categories

*: Statistically significant at *p* ≤ 0.05

### Logistic regression analysis

In multivariate logistic regression, difficulty in getting medications (adjusted OR, 1.012; 0.815–1.810, *p* = 0.040), non-compliance to treatment (adjusted OR, 0.579; 95%CI, 0.418–0.804, *p* = 0.001), medication overuse (adjusted OR, 11.603; 8.403–16.023, *p* < 0.001), sleep disturbance (adjusted OR, 2.113; 1.375–3.248, *p* = 0.001), eating habits disturbance (adjusted OR,1.702; 1.178–2.459, *p* = 0.005), working during pandemic (adjusted OR, 1.356; 1.169–2.368, *p* = 0.005), and lack of communication with treating physician (adjusted OR, 1.983; 0.898–2.730, *p* = 0.001) were still found to be significantly associated with increase in migraine frequency.

Moreover, the same aforementioned variables, in addition to female gender (adjusted OR, 0.643; 0.419–0.986, *p* = 0.043), shorter disease duration (adjusted OR, 0.978; 0.960–0.996, *p* = 0.018) and symptoms of anxiety and/or depression (adjusted OR, 1.558; 1.043–2.329, *p* = 0.030), remained statistically significant for migraine severity worsening. However, use of traditional medicine and lack of regular exercise were not found to be independent risk factors for increase in both migraine frequency or severity in the multivariate model. The detailed results of multivariate analysis are shown in Table 3.

**Table 3:** Multivariate logistic analysis to identify independent variables affecting increase in migraine frequency and severity (n = 1018)

Variables	Increase in migraine frequency		Increase in migraine severity	
	AOR (95%CI)	***p***-value	AOR (95%CI)	***p***-value
**Gender (female)**	0.769 (0.491–1.203)	0.250	0.643 (0.419–0.986)	0.043^*^
**Disease onset**	0.984 (0.965–1.003)	0.089	0.978 (0.960–0.996)	0.018^*^
**Difficulty in getting medications**	1.012 (0.715–1.810)	0.040^*^	1.271 (0.872–1.853)	0.002^*^
**Non-compliance to treatment**	0.579 (0.418–0.804)	0.001^*^	1.655 (1.204–2.274)	0.002^*^
**Medication overuse**	11.603 (8.403–16.023)	< 0.001^*^	8.179 (5.963–11.218)	< 0.001^*^
**Sleep disturbance**	2.113 (1.375–3.248)	0.001^*^	1.562 (1.041–2.344)	0.031^*^
**Eating habits disturbance**	1.702 (1.178–2.459)	0.005^*^	1.778 (1.247–2.536)	0.001^*^
**Symptoms of anxiety and/or depression**	1.257 (0.823–1.918)	0.290	1.558 (1.043–2.329)	0.030^*^
**Use of traditional medicine**	1.465 (1.049–2.045)	0.071	1.361 (0.984–1.882)	0.063
**Working during pandemic**	1.356 (1.169–2.368)	0.005^*^	1.241 (0.906–1.699)	0.001^*^
**Lack of communication with treating physician**	1.983 (0.898–2.730)	0.001^*^	1.776 (0.898–2.938)	0.001^*^
**Lack of regular exercise**	1.257 (0.898–1.761)	0.183	1.223 (0.898–1.412)	0.234

AOR: Adjusted odds ratio

CI: Confidence interval

*: Statistically significant at *p* ≤ 0.05

## Discussion

In this survey, COVID-19 pandemic had a negative impact on patients with migraine. The number of respondents was high, compared to recent reported numbers of patients with migraine in Kuwait [4]. More than half of them experienced increase in migraine frequency and severity, in comparison to pre-pandemic period. This was understandably accompanied by overuse of analgesics and acute migraine treatments, however, only 22.5% reported visiting ED for acute management, probably for fear of contracting the virus.

In light of survey data, female gender, shorter disease duration, having difficulty in getting medications, lack of communication with treating neurologist, non-compliance to treatment, and working during the pandemic, had a statistically significant correlation with worsening of migraine symptoms. Moreover, symptoms of anxiety and/or depression, disturbance of sleep and eating habits, lack of regular exercise, overuse of analgesics and use of traditional medicine (TM), were significantly higher in this group. However, there was no statistically significant correlation with age, occupation, educational level, marital status, and screen time exposure.

The stress-related psychosocial impact of the pandemic is evident and complex. Several recent studies documented this impact on patients with chronic diseases [8, 9]. A global survey from 47 countries reported worsening of the mental health of 80% of patients with chronic diseases during COVID-19 outbreak [10]. Another recent study from China on 144 migraine patients showed more severe psychological distress in those patients during the pandemic, in comparison to normal population. High frequency of migraine attacks and attention paid to COVID-19 media coverage were identified as risk factors [11].

Most respondents experienced significant life changes in such a short period of time. These include changes in their sleep and eating habits, financial and work-related concerns, cancellation of activities, increase in screen time exposure, in addition to the general worries associated with the pandemic such as contagion, lockdown, social isolation and information overdose.

There is enough evidence that stress is the most frequent trigger of migraine attacks as reported by patients [12, 13]. COVID-19 pandemic, as a global health crisis, is perceived as a major stressful event [14]. Fear of COVID-19 infection was the most common concern reported by patients (56.1%), followed by fear of headache worsening (43.7%). In a recent study from Spain, 41% of participants reported feeling moderately to severely stressed during the pandemic, with 41% having depressive symptoms, and 25% having mild to severe levels of anxiety [15]. Similar to our findings, female gender and young age felt the strongest negative impact. In our survey, headache worsening was almost perceived as sinister as contracting a potentially fatal virus.

Furthermore, migraine is strongly linked to both depression and anxiety. In literature, 50–60% of patients with migraine were found to be anxious and/or depressed, and around 40% complained of sleep disturbance [16, 17]. In our study, 79.5% and 78.1% were found to have symptoms of anxiety and/or depression, and sleep disturbance, respectively. This was higher than the overall prevalence in the general population amid the pandemic, as shown in a recent Chinese cross-sectional survey on 7236 participants. Prevalence of anxiety, depressive symptoms, and sleep disturbance was 35.1%, 20.1%, and 18.2%, respectively [18]. This high prevalence in our survey can be explained in the light of higher levels of perceived stress, in addition to worsening of migraine symptoms. Migraine attacks themselves can act as a stressor, creating a vicious circle that increases both migraine severity and frequency [12].

More than 60% of surveyed patients failed to communicate with their neurologists during the lockdown. A recent survey of 155 countries conducted by the World Health Organization (WHO) found that nearly half of the patients with chronic diseases failed to receive their regular medical care and medications since COVID-19 pandemic began [19]. We believe that poor communication with the healthcare system had forced patients to attempt self-management, by overusing analgesics or using TM, which was not correlated with satisfactory headache care.

Around 40% of respondents reported using TM during the pandemic. The most commonly used therapies were herbal remedies, head banding, head massage, ice packs, supplements and relaxing techniques. However, only a minority of respondents underwent procedural TM such as blood cupping (Hijama), which is the most common TM for headache in Arab countries. The use of TM in migraine patients under medical treatment was 30.1% in Kuwait [20] and 31.4% in Italy [21]. The higher use of TM can be explained as a way of self-treatment with increasing headaches and decrease access to medical care. As a result, 10.3% of respondents transformed to chronic migraine during lockdown. Studies estimate that about 2.5% of patients with episodic migraine will transform to chronic migraine each year [22]. The high levels of stress they endured during lockdown, in addition to the overuse of acute migraine medications, are known risk factors for migraine chronification [23, 24].

Surprisingly, 16% of respondents reported reduction in headache days per month. This could be explained by the reduction of work-related pressure, especially if patients were not worried about losing their job, in addition to possible improvement in sleep quality and eating habits. This is similar to a recent study from Italy [25] that reported fewer migraine attacks and lesser pain in 49 patients with migraine. The authors attributed the findings to less triggers as a result of less work.

Only 4% of respondents developed COVID-19 infection in our survey, and around two-thirds of them reported worsening of their headaches. In recent studies, headache seems to be the 5th most frequent COVID-19 symptom and the most frequent neurologic complaint, occurring in around 6.5% [26] to 12% [27] of patients. Approximately one third of COVID-19-associated headaches occurred in patients with previous history of migraine [28]. It is worth mentioning that in rare occasions, headaches in those patients can be secondary to viral infection of the central nervous system (CNS). Careful evaluation and recognition of “red flags”, especially with refractory or persistent headaches, is warranted [29, 30].

Migraine patients who depend on “onabotulinumtoxinA” in particular had been vulnerable to inadequate quality care during the pandemic. Two-thirds of them reported negative impact of cancellation or postponing of their injections. In the absence of therapeutic procedures, other preventive therapies such as self-injectable monoclonal antibodies, neuromodulation devices and oral corticosteroids could be tried. There is no evidence that the use of nonsteroidal anti-inflammatory drugs (NSAIDS) can worsen COVID-19 clinical course, thus it could be used appropriately [2, 31]. However, we stress that paracetamol (acetaminophen) can be used as a first choice in headache treatment, given its safety and better tolerability [32].

## Recommendations

The ongoing threat of COVID-19, or other future pandemics, will require long-term strategies to deliver quality care for patients with migraine. Telemedicine has become an essential modality for headache specialists [33, 34]. Globally 58% of countries are now using telemedicine to replace in-person consultations [35]. In Kuwait, only 2% used telemedicine clinics and 19.4% reported using telephone calls or social media. We believe that communication with the treating neurologist, regardless the method itself, is of utmost importance to good outcome. Other strategies include preparing “rescue care” plans, home delivery of medications, and self-administered therapies, to keep patients away from ED and limiting their exposure to COVID-19. Patients with migraine should be educated and advised to stick to their previous routine as much as possible. Maintaining regular sleep hygiene, healthy diet, and good hydration are important, in addition to avoiding information overload, self-isolation and excess screen time exposure. Psychological distress among patients with migraine should not be overlooked, and must be properly managed.

### Limitations

This survey study had some limitations. First, part of the respondents self-selected themselves into the study via social media, which may be prone to selection bias. However, a significant portion were recruited from the outpatient headache clinic, and only patients who received a medical diagnosis of migraine prior to the pandemic were included. Not limiting the survey to patients from a single-center can help generalizability of the findings to a wider patient population.

Secondly, the responses were quite unbalanced for sex and age, as there was a tendency for our respondents to be young with female predominance. This might limit the generalizability of our results; however, migraine is known to be more common in females, with the highest prevalence in the 20–50 age group. Also, younger population usually constitute the majority of social media users, which was used for distributing this survey. Further, we are aware that using social media for sampling is not the best modality and not immune to several biases, however, our aim was to include the highest number of migraine patients in Kuwait in a time of movement restrictions.

Finally, being an observational online survey study, the establishment of a direct causal relationship can be difficult. However, we present a “real-world” evidence of the impact of an unprecedented global crisis on patients with migraine, with an overall adequate sample size. This will help better understanding of their needs and sufferings, and to help planning and implementing future strategies.

## Conclusions

Our study provides “real-world” data regarding the negative impact of COVID-19 pandemic on migraine. The majority of respondents struggled with increase in migraine frequency and severity. Patients attempted self-management by overusing analgesics, and using TM. Around 10% of patients transformed to chronic migraine during lockdown.

We identified the most relevant risk factors that influenced worsening, which included disruption of sleep and dietary habits, associated symptoms of anxiety and depression, lack of communication with treating neurologist, non-compliance to treatment, and working during the pandemic. Considering that this current health crisis will most likely have long lasting effects, it is important to validate and implement long-term strategies for those patients, in order to continue to receive quality headache care.

## Data Availability

The data generated for this study are available on request to the corresponding author.

## Abbreviations

COVID-19: Coronavirus Disease 2019
SARS-CoV-2: Severe acute respiratory syndrome coronavirus
ED: Emergency Department
TM: Traditional medicine
WHO: World Health Organization
CNS: Central nervous system
NSAIDS: Non-steroidal anti-inflammatory drugs
